# A Generic Framework for Depth Reconstruction Enhancement

**DOI:** 10.3390/jimaging8050138

**Published:** 2022-05-16

**Authors:** Hendrik Sommerhoff, Andreas Kolb

**Affiliations:** Computer Graphics Group, Center for Sensor Systems (ZESS), University of Siegen, Hölderlinstraße 3, 57076 Siegen, Germany; andreas.kolb@uni-siegen.de

**Keywords:** deep learning, depth image, denoising, super resolution, deblurring

## Abstract

We propose a generic depth-refinement scheme based on GeoNet, a recent deep-learning approach for predicting depth and normals from a single color image, and extend it to be applied to any depth reconstruction task such as super resolution, denoising and deblurring, as long as the task includes a depth output. Our approach utilizes a tight coupling of the inherent geometric relationship between depth and normal maps to guide a neural network. In contrast to GeoNet, we do not utilize the original input information to the backbone reconstruction task, which leads to a generic application of our network structure. Our approach first learns a high-quality normal map from the depth image generated by the backbone method and then uses this normal map to refine the initial depth image jointly with the learned normal map. This is motivated by the fact that it is hard for neural networks to learn direct mapping between depth and normal maps without explicit geometric constraints. We show the efficiency of our method on the exemplary inverse depth-image reconstruction tasks of denoising, super resolution and removal of motion blur.

## 1. Introduction

High-quality depth maps are required in a wide variety of tasks in computer vision and graphics, such as RGB-D scene reconstruction [1,2], augmented reality [3,4,5] and autonomous driving [6,7,8]. Compared to standard RGB-sensors, depth sensors often produce noisy images, which makes depth-reconstruction tasks especially challenging, since every task also has to account for the different task-specific depth uncertainties or deficiencies. Some classes of sensors have types of artifacts that are not common in that form for typical color sensors. For example, artifacts from motion relative to the camera are a particular problem for Time-of-Flight (ToF) cameras because they capture multiple phase images in sequence. Solutions for these problems require specialized algorithms such as the ones outlined in [9].

Even though approaches that are well known in the realm of color-image enhancement, such as energy minimization methods or deep learning, can often be translated one-to-one to depth enhancement tasks, usually by just interpreting the depths as grayscale values. This fails to incorporate the inherent geometric structure of depth maps. While research on depth-only enhancement exists [10,11], a majority of recent work has focused on some form of intensity or RGB-guided depth enhancement, e.g., for super resolution [12,13,14,15], denoising [16,17] or motion blur removal [18,19]. While this greatly improves the quality of the resultant depth images, these additional RGB sensors are not always available. Moreover, none of the examples above explicitly incorporate surface normal information, which is geometrically tightly linked to the depth map information. However, in the area of depth estimation from a single RGB-image, there have recently been works that not only produce normal maps as an additional output, but also successfully use them to enhance the quality of the final depth map [20,21,22,23,24]. Most notably, Qi et al. [20,21] introduce the GeoNet/GeoNet++ network architecture to estimate a depth and a normal map from a single RGB image. Their approach toggles between depth-to-normal that utilizes a least squares approach, and normal-to-depth estimation based on kernel regression to enforce geometric consistency between the two domains. Their approach can be seen as a weak coupling between normals and depth, as the two stages operate independently. Still, GeoNet++ outperforms standard CNN approaches that learn direct mapping between depth and surface normals, both in terms of accuracy and normal-depth consistency. In an ablation, the authors show that CNNs have problems to learn a direct mapping between depth and surface normals in general [21]. Since it is already hard to learn this mapping in a supervised setting with normals as output, we hypothesize that neural networks also have difficulties including surface normal information in their latent representations without explicit geometric constraints.

In this paper, we develop a generic depth refinement scheme that takes surface normals into account but makes no assumptions about the specific task that is to be solved, except that the output is a depth map. Based on the GeoNet/GeoNet++ concept, our approach computes high-quality normal maps in an intermediate step, which are then used to refine an initial depth estimate provided by the backbone method. Contrary to GeoNet/GeoNet++, we do not utilize the original input to the backbone method, making our approach generic to many existing reconstruction methods. Moreover, we use a tighter coupling between the depth and the normal domain by linking both stages using skip connections, making full normal and depth information available in both stages.

Our experiments show that this approach improves the quality compared to existing methods in a variety of different tasks, namely depth-only super-resolution, RGB-guided super-resolution, additive Gaussian noise removal and deblurring.

## 2. Related Work

In this section, we will give a brief overview of research in different areas of depth reconstruction. We will roughly split the methods into classical variational methods and deep-learning-based methods.

Specialized variational and classical non-learning-based approaches for depth reconstruction generally aim to improve depth data with additional sensor data like color images. Huhle et al. [25] use a non-local means (NLM) approach to remove outliers from depth data by computing an additional color-based weight in their NLM formulation. Ferstl et al. [26] use a variational approach to compute higher-resolution depth images with the help of already high resolution intensity images. Some approaches specialize in specific sensor types: Shen and Cheung [27] introduce a probabilistic model using a Markov random field for denoising and completing depth maps from structured light sensors. Another work on structured light sensors was presented by Fu et al. [28], who specifically target the spatiotemporal denoising of the Microsoft Kinect camera.

In recent years, like in any other field of computer vision and graphics, there has been substantial amounts of deep-learning research for depth reconstruction. Sterzentsenko et al. [16] used self-supervision to train a deep autoencoder to combat the lack of real world datasets with noise-free ground truth depths. The work from Tourani et al. [18] deals with the removal of motion artifacts from rolling shutters, which are common in structured sensors such as the Kinect. Li et al. [19] use a two-branched CNN to simultaneously remove motion blur from a color and a depth image. The problem of depth-only super-resolution, i.e., without additional color data, was tackled by Li et al. [11] who extend ideas from deep Laplacian pyramid networks [29], which were originally proposed for RGB super-resolution, to depth. They put their work into the context of 3D reconstruction, which they show can greatly benefit from higher-resolution depth-maps. Research in the area of color-guided depth super-resolution is more widespread. Zhao et al. [30] jointly upscale depth and color images by using a generative adversarial neural network (GAN). Another deep-learning-based approach was proposed by Kim et al. [13] in the shape of deformable kernel networks (DKN) for joint image filtering. Apart from guided depth image super-resolution their approach can also be applied to saliency map upsamling, cross modality image restoration and texture removal. Recently Tang et al. [14], inspired by progress in neural implicit representations, introduced joint implicit image functions (JIIF) and interpreted the problem of guided depth super-resolution as a neural implicit interpolation task. Another recent deep-learning-based approach is by Zhong et al. [31] who used an attention-based network design to fuse the most important features from depth and color images and then used those features to guide an upscaling network. There have also been hybrid methods which combine classical approaches with deep-learning techniques, e.g. Riegler et al. [10] who combined traditional variational methods with a deep neural network to improve the accuracy of depth super-resolution without the need for additional color sensors.

Even though the experiments in our manuscript do not include depth prediction from single-color tasks, works from this field that use explicit surface normal information are also related to our approach. Apart from GeoNet by [20,21], which our work directly extends and we will discuss in more detail in the upcoming sections, we will list some other research in that direction. Eigen and Fergus [22] tackle the task of depth and normal prediction and semantic segmentation from RGB images in a single deep neural network. Xu et al. [23] first predict initial depth, surface normal, semantic segmentation and contour maps and then fuse them into a final depth-map. However in both of these works, there is no enforcement of consistency between the predicted normal and depth images. A more tightly coupled approach was proposed by Wang et al. [24], who introduced an orthogonal compatibility constraint between normals and surface points that lie in a common planar region. However, their computations are very costly and the method might fail in non-planar regions of the scene.

## 3. Method

In this section, we introduce our generalized depth-enhancement framework for arbitrary image-reconstruction tasks. First, we will briefly review the main ideas from Qi et al. [20,21] in Section 3.1. In Section 3.2, we introduce our general depth-enhancement network. Finally, we discuss the loss functions used in Section 3.3 and implementation details in Section 3.4.

### 3.1. GeoNet

Originally, GeoNet is a method for estimating a normal and a depth map from a single RGB image. In the following explanations of GeoNet, it is assumed that initial normal and depth estimates, by whichever means, e.g., another CNN, have already been computed. The initial normal at pixel *i* is denoted as $n_{i}^{initial}$ and the initial depth at pixel *i* as $z_{i}^{initial}$. Further following the notation of Qi et al. [20,21], we denote pixel coordinates as $\left(u_{i},v_{i}\right)$ and corresponding 3D coordinates as $\left(x_{i},y_{i},z_{i}\right)$. The mapping between the the two is determined by the perspective projection equations

(1) $$\begin{array}{cc} x_{i} & =\left(u_{i}-c_{x}\right)z_{i}/f_{x}\\ y_{i} & =\left(v_{i}-c_{y}\right)z_{i}/f_{y} \end{array}$$

where $f_{.}$ and $c_{.}$ are the intrinsic camera parameters.

The main idea of [20] is now to refine the initial normal map by using the geometric constraints given by the depth map, and vice versa. This is motivated by the fact that both representations have an inherent geometric relationship with each other that is hard to learn directly through a network. We will now discuss both paths—depth refinement using normals and normal refinement using depth—separately.

#### 3.1.1. Normal Refinement

To refine the initial normal map $n^{initial}$, first, an additional normal map that is consistent with the initial depth-map is computed. To avoid confusion, we will denote normals from this auxiliary normal map as $n_{i}^{depth}$. By using the assumption that surface points in a local neighborhood approximately lie on the same plane, $n_{i}^{depth}$ can be computed from $z_{i}^{initial}$ by first projecting the local neighborhood back into 3D using Equation (1) and then computing the normal using least squares. The neighborhood of size β around *i* is defined as

(2) $$N_{i}=\left\{\left(x_{j},y_{j},z_{j}\right)\left|\right|u_{i}-u_{j}\left|<\beta ,\right|v_{i}-v_{j}\left|<\beta ,\right|z_{i}-z_{j}|<\gamma z_{i}\right\},$$

where γ is a parameter to filter out depths which deviate too much from the center depth. Writing the points of this neighborhood into a matrix

(3) $$A=\left[\begin{array}{ccc} x_{1} & y_{1} & z_{1}\\ x_{2} & y_{2} & z_{2}\\ \vdots & \vdots & \vdots \\ x_{K} & y_{K} & z_{K} \end{array}\right]\in R^{K\times 3},$$

enables the calculation of the normals as the least squares solution

(4) $$n_{i}^{depth}=\frac{{\left(A^{⊤}A\right)}^{-1}A^{⊤}1}{\left|\right|{\left(A^{⊤}A\right)}^{-1}A^{⊤}1\left|\right|}.$$

Here 1 is the K-dimensional constant vector with only 1 s. Since this normal is prone to noise, it is further refined by a residual network that also takes $n^{initial}$ as input. In [21], it is defined as

(5) $$n^{final}=N_{2}\left(\left(N_{1}\left(n^{depth}\right)+n^{depth}\right)\circ n^{initial}\right),$$

where $N_{1}$ and $N_{2}$ are CNNs and ∘ means concatenation along the channel dimension. The output of this network $n_{i}^{final}$ is the refined normal map. In our experiments we additionally tried to replace the least squares normals with cross product normals which unfortunately resulted in very high noise and unsatisfactory results. All methods in this paper therefore use least squares normals, as seen in [20] as described above.

#### 3.1.2. Depth Refinement

Analogous to the previous section, the first step is to compute a depth map $z^{normal}$ that is consistent with the initial normal map. The assumption is the same: points in a close neighborhood lie on the same plane. The neighborhood around pixel *i* is defined as

(6) $$M_{i}=\left\{\left(x_{j},y_{j},z_{j}\right)\left|\right|u_{i}-u_{j}\left|<\beta ,\right|v_{i}-v_{j}|<\beta ,n_{j}^{⊤}n_{i}>\alpha \right\}.$$

Instead of filtering out large depth deviations, normals with a large angular difference to the center normal are filtered out.

Given only the center depth, the depth for each point in the neighborhood can now be estimated as

(7) $$z_{ji}^{\prime }=\frac{n_{jx}x_{j}+n_{jy}y_{j}+n_{jz}z_{j}}{\left(u_{i}-c_{x}\right)n_{jx}/f_{x}+\left(v_{i}-c_{y}\right)n_{jy}/f_{y}+n_{jz}}.$$

These depth estimates are then aggregated by weighting them with the angular difference of their normal to the center normal by kernel regression

(8) $$z_{i}^{depth}=\frac{\sum_{j}\left(n_{j}^{⊤}n_{i}\right)z_{ji}^{\prime }}{\sum_{j}\left(n_{j}^{⊤}n_{i}\right)}.$$

Again, these rough estimates are further refined with a CNN

(9) $$z^{final}=N_{3}\left(z^{depth}\circ z^{initial}\right).$$

Note that all operations above, particularly computing least squares solutions and kernel regression, are differentiable, which means all networks, including the upstream RGB-to-depth network, can be trained end-to-end.

### 3.2. General Depth Enhancement Network

We will now explain how we extend the ideas from GeoNet [21] from its RGB-to-depth estimation task to arbitrary depth-to-depth refinement tasks. We assume that we have some generic algorithm G (such as a neural network) that maps the input *x* (e.g., a low-resolution depth-map) to an initial depth-map estimate of its specific task (such as super-resolution).

(10) $$z^{initial}=G\left(x\right).$$

We refer to G as *backbone (network)*, but note that, despite our experiments only including neural networks as choices for G, we make no assumptions on the structure or differentiability, i.e., it could in theory also be a classical image-reconstruction method such as non-local means or energy minimization.

Unlike GeoNet, which also requires an additional backbone for initial normal computation, we only require a generic backbone that maps *x* to an initial depth estimate. Moreover, our approach does not utilize the original input data *x* to the backbone network G, making it independent from the underlying refinement task. Instead of having the two independent depth and normal refinement branches, we propose a single sequential refinement scheme in which we first compute a high-quality normal map from the initial depth-map and then use this normal map to refine the depth map again.

We use Equation (4) to calculate a rough normal estimate $n^{depth}$. Unlike in Equation (5) we also concatenate the initial depth to the refinement network and add additional skip connections. Compared to GeoNet++, these skip connections enforce a tighter handling of depth and normal information in both stages.

(11) $$n^{final}=N_{2}\left(\left(N_{1}\left(n^{depth}\right)+n^{depth}\right)\circ z^{initial}\right)+n^{depth}.$$

These normals are then used to refine the depth map again. The idea here is that first guiding the network to learn accurate normals might help it to find geometric structure that it would have otherwise missed.

We use Equations (7) and (8) to compute a intermediary depth estimation $z^{normal}$ which is further refined into our final result by applying a CNN. Again, we add additional skip connections and concatenate the normal map to improve results:

(12) $$z^{final}=N_{3}\left(z^{normal}\circ n^{final}\circ z^{initial}\right)+z^{initial}.$$

**Figure 1:**
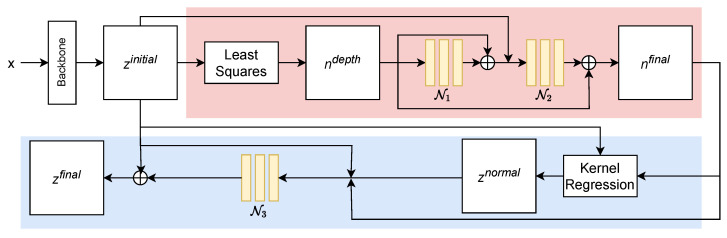
The proposed network architecture. Red—normal refinement module; blue—depth-refinement module; yellow—CNNs with learnable parameters.

The overall architecture of our scheme is visualized in Figure 1. Most parts of the architecture are fixed weight and not learnable, which makes the training converge quickly. The concrete implementation of the CNNs like the number of layers of kernel sizes will be discussed in Section 3.4.

### 3.3. Loss Functions

Analogously to GeoNet, every operation from the initial depth estimate $z^{initial}$ to the refined estimate $z^{final}$ is differentiable. This means all networks (including the backbone, if it is also a neural network) can potentially be trained in an end-to-end fashion. However in the experiments in this paper, to showcase the generality of our approach, we pretrain the backbones and freeze their weights before training our remaining network. We compute loss functions on the intermediate results and sum up the individual losses to the total loss function $l=l_{normal}+l_{depth}$. More specifically, our normal loss function is the same as in [21]:

(13) $$l_{normal}=\frac{1}{K}\left(\sum_{i}\left|\right|n_{i}^{final}-n_{i}^{gt}{\left|\right|}_{2}^{2}+\lambda \sum_{i}\left|\right|n_{i}^{depth}-n_{i}^{gt}{\left|\right|}_{2}^{2}\right).$$

For the depth loss, we make a few modifications. We do not include a loss function on the direct output of the backbone, since its weights are frozen. Instead, we also compute a loss on $z^{normal}$. Even though there is only the kernel regression step with no learnable parameters between the computations of $n^{final}$ and $z^{normal}$, we found in our experiments that it is still beneficial to have this additional loss function to pass gradients to the upstream networks. We use the Charbonnier loss-function [32] instead of L2 loss:

(14) $$l_{depth}=\frac{1}{K}\left(\sum_{i}\sqrt{{\left(z_{i}^{final}-z_{i}^{gt}\right)}^{2}+\epsilon }+\eta \sum_{i}\sqrt{{\left(z_{i}^{normal}-z_{i}^{gt}\right)}^{2}+\epsilon }\right).$$

To pretrain the backbone networks, we use the same Charbonnier loss-function (here of course only with one summand).

Note that even though we need ground truth normal maps during training, at no point do we need normal map inputs during inference. This allows us to put our network on top of any arbitrary backbone as long as it outputs depth images.

### 3.4. Implementation Details

We use the same network architecture on top of each backbone. Each CNN in our scheme (see Figure 1) consists of just four convolutional layers with kernel size 3 and hidden dimension 64, which results in 235K additional learnable parameters. Table 1 shows the parameter and runtime overhead of our network compared to different backbones. We use ReLUs as our activation functions. We choose η=0.5 and $\lambda =10^{-3}$ for the loss-weighting hyperparameters and $\epsilon =10^{-6}$ as the parameter of the Charbonnier loss. We set the neighborhood size to 9×9 in Equation (2) and Equation (6) and choose γ=0.05 and α=0.95. We center crop images to a size of 256×256, randomly flip images along the vertical axis for data augmentation and train with a batch size of 16. As mentioned before, we freeze the weights of the backbones in all our experiments. In general, only 2–3 additional epochs are needed for our model to converge.

## 4. Training and Tasks

We demonstrate the effectiveness of our method on a variety of classical image-reconstruction tasks. To show the general nature of our approach, we add it and compare it to several different state-of-the-art backbone networks. All backbone networks were trained from scratch using code provided by the authors, using the training data provided by Qi et al. [20]. The dataset is based on the NYU v2 dataset [33] and contains 30,816 frames with real-world depth and color images taken with a Microsoft Kinect, as well as high-quality normal maps that we used as ground truth. For more details on this training set, refer to [20]. The input to the networks was simulated from the ground truth images with the respective forward operators of the different tasks and will be further detailed in the following sections.

### 4.1. Denoising

The first task we used for comparison was the removal of additive Gaussian noise with known variance. We compared it against the two state-of-the-art deep-learning methods DnCNN [34] and the attention-based ADNet [35]. We added randomly sampled Gaussian noise with a moderate standard deviation of 0.5 m to our ground truth depth images and trained the networks with default parameters.

### 4.2. Deblurring

We convolved the ground truth depth with a 25×25 blur kernel that contained zeros everywhere except on the main diagonal, where it was constant 1/25. This roughly simulated motion blur of a far-away scene when the camera was rotated diagonally from the top left to the bottom right. We used a 17-layer ResNet as backbone, with a similar architecture to DnCNN [34].

### 4.3. Super-Resolution

We covered methods from both depth-only super-resolution as well as color-guided super-resolution in our experiments. For the former, we used DLapSRN [11], which itself is based on Laplacian pyramid networks [29]. Our backbone for color-guided super-resolution is the recent deformable kernel network (DKN) [13]. We used bilinear filtering to sub-sample the ground truth depth images to a factor of 1/4. Again, we trained the networks with default parameters until convergence.

In order to gauge how our network deals with inputs of lower quality, we also trained it together with a simple bilinear interpolation backend. This also showcases how our method is not limited to learning-based backends.

## 5. Results

We evaluated the different methods on a separate 654 image subset of the common benchmark dataset NYU v2 [33], which is often used to evaluate super-resolution tasks [10,13,14]. To the best of our knowledge, there are no such commonly used benchmark datasets for depth-map Gaussian denoising and deblurring. For this reason, we evaluated all tasks on the same datasets. Quantitative results can be seen in Table 2. Our add-on network consistently improved the results of all backbone networks both in terms of root-mean-square-error (RMSE) and mean-absolute-error (MAE). Since we used the exact same backbone as a stand-alone network in the comparison, this improvement has to be a result of our depth-refinement scheme. The improvements of our network ranged from 3% for ADNet to 20% for DLapSRN in terms of average RMSE and from 6% to 20% in terms of MAE. This discrepancy could be explained with the quite challenging noise level of 0.5 m in our denoising experiments. Since the outputs of the backbones still included many defects, our initial normal computation could output low-quality normals that are not as helpful to the depth refinement network. Note that our add-on-like approach with a skip connection between the backbone output and the final result helps our method to be at least of the same quality as the backbone output, because in the worst case the network could just learn to output the initial depth-map. In terms of the structural similarity index (SSIM) [36], the deblurring experiment is slightly worse than the baseline, but in general the margins are lower, with the exception of the DnCNN experiments. Note that we did not explicitly optimize the networks for perceptual quality.

To show that our network is able to generalize to new datasets, we also evaluated 30 images of the Middlebury stereo dataset [37] without fine tuning our networks. The Middlebury dataset contains pixel disparity images which other authors [10,11,13,14] directly interpret as depth values before feeding them into their method. Since we needed to reproject depth values in order to compute our initial normal maps, we first needed to convert the disparity images into real depth images before inputting them into the backbones. To make our results comparable to other methods, we converted the final depth-maps back into disparity values before computing evaluation metrics. The results in Table 3 show that our network consistently outperformed the baseline methods. Since the Middlebury uses stereo images, as opposed to the training set, which uses structured light [33], we conclude that all tested networks can generalize to different types of sensors.

**Figure 2:**
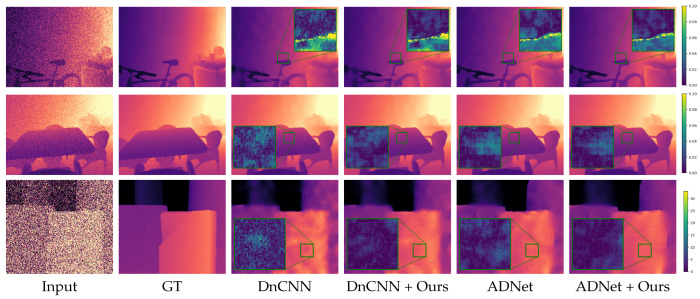
Denoising results. Insets show absolute difference to ground truth. The first two rows are examples from the NYU v2 [33] test set and the third row from the Middlebury dataset [37]. Note that while we, like other authors, show input and result of the Middlebury example as pixel disparity values, we add the noise to the converted depth maps.

**Figure 3:**
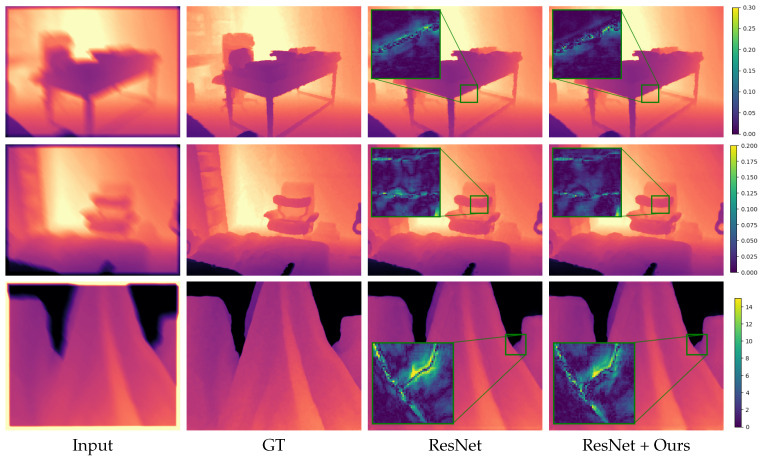
Deblurring results. Insets show absolute difference to ground truth. The first two rows are examples from the NYU v2 [33] test set and the third row from the Middlebury dataset [37].

**Figure 4:**
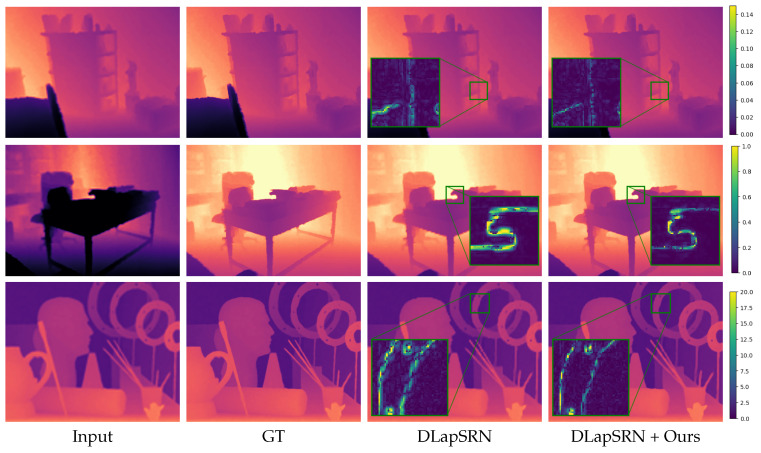
Depth-only super-resolution results. The input is upscaled for visualization purposes. Insets show absolute difference to ground truth. The first two rows are examples from the NYU v2 [33] test set and the third row from the Middlebury dataset [37].

**Figure 5:**
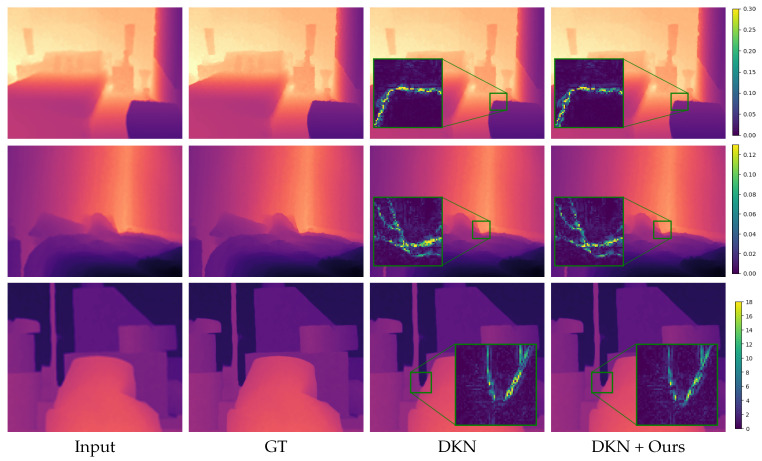
RGB-guided super-resolution results. The input is upscaled for visualization purposes. Insets show absolute difference to ground truth. The first two rows are examples from the NYU v2 [33] test set and the third row from the Middlebury dataset [37].

We show qualitative results in Figure 2, Figure 3, Figure 4 and Figure 5. For better visualization, we show pixel-wise absolute difference to ground truth depth inside the insets. The areas of the highest improvement differ between tasks. Our method improved the denoising backbones mostly in planar regions (Figure 2). We assume that here, our windowed least-squares normal computation acted as an additional low-pass filter. Nevertheless, sharp edges were still preserved by our network. In contrast, the deblurring (Figure 3) and super-resolution backbones (Figure 4 and Figure 5) already output high-quality planar regions and the improvements of our network were predominantly located at the edges. The differences in results for RGB-guided super-resolution in Figure 5 are more subtle. DKN can already achieve very sharp edges by utilizing color-image information, and our method seemed to mostly improve some outliers at those edges.

**Figure 6:**
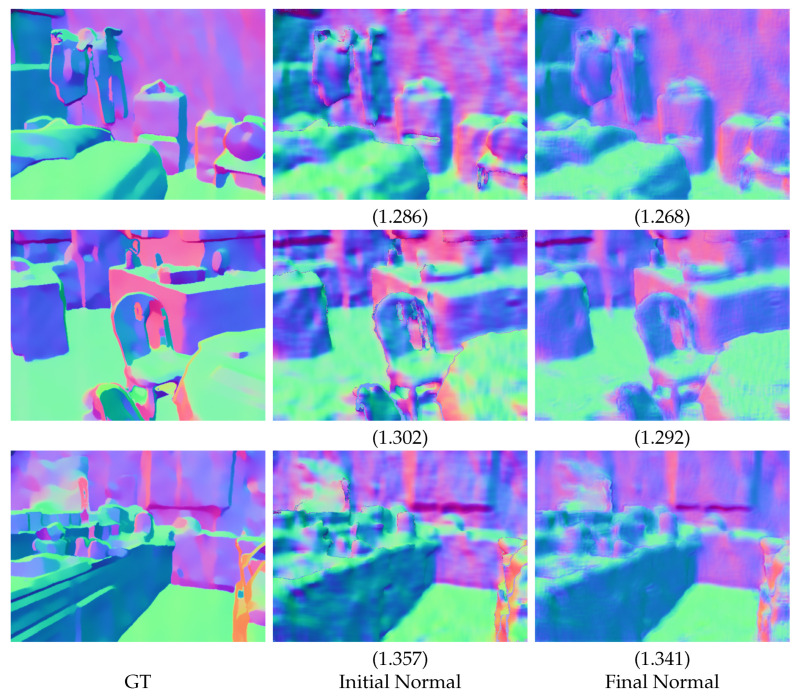
Visualization of normal map quality on three scenes of the NYU v2 dataset using the ADNet backbone. Values directly under the image are mean angular error to ground truth.

To verify our normal refinement module, we show exemplary normal map visualizations from our denoising results using the ADNet backbone in Figure 6. As suggested above, the normal computation for the denoising task is more challenging than for the other tasks. Our final normal maps are less noisy than the normal map that was directly computed with least squares. Especially at the edges, the initial normals show high levels of noise. They are also better in terms of mean angular error. Note that we focused more on high-quality depth-maps when we fine tuned our hyperparameters, and in general, treat the normal maps as auxiliary data to improve those depth maps. Since our loss function is a weighted average of depth and normal loss and depth information can also propagate through the normal refinement module, the network could learn to output slightly lower-quality normals if it, in turn, helps to improve the depth map and lead to a lower local minimum.

## 6. Conclusions

In this paper, we have introduced a generic depth enhancement framework for a potentially wide variety of depth-reconstruction tasks. Our method is able to improve on several state-of-the-art deep-learning-based methods by adding just a few additional learnable parameters. Our approach has the nice side effect that it also computes high-quality normal maps that can be utilized in some tasks, e.g., 3D reconstruction.

There are multiple possible directions for future work. Since we froze the weights of all pretrained backbone networks while training our depth-enhancement network, it would be interesting to see if improvements could be made by training them in tandem. We also speculate that our model can be rather easily used for transfer learning because the output of the backbones is already very similar. Another possible direction is to apply our depth-enhancement scheme iteratively, similar to the considerations from Qi et al. in the GeoNet++ paper [21]. In essence, the enhanced depth-map can again be used to compute a higher-quality normal map, which in turn can be used to get an even more improved depth-map, and so on.

## Figures and Tables

**Table 1:** Learnable parameters and timings of the networks in our experiments. Timings were measured using a NVIDIA Tesla V100 and AMD EPYC 7452 on a single 640×480 image (output size) from the NYU v2 dataset [33].

**Network**	DnCNN	ADNet	ResNet	DLapSRN	DKN	+Ours
**Parameters**	556K	519K	556K	435K	1.4M	+235K
**Time (ms)**	26	28	26	10	126	+34

**Table 2:** Quantitative comparison of depth-map reconstruction for different tasks on the NYU v2 dataset [33]. Values are given in centimeters (RMSE and MAE) and averaged over all test set images.

Task	Method	RMSE ↓	MAE ↓	SSIM ↑
Denoising	DnCNN [34]	4.07	2.84	0.9663
	DnCNN [34] + Ours	3.81	2.57	**0.9757**
	ADNet [35]	3.64	2.47	0.9730
	ADNet [35] + Ours	**3.55**	**2.34**	0.9743
Deblurring	ResNet [38]	3.14	2.14	**0.9897**
	ResNet [38] + Ours	**2.97**	**2.00**	0.9896
Super-resolution (Depth only)	Bilinear	3.63	1.09	0.9821
	Bilinear + Ours	3.07	0.93	0.9849
	DLapSRN [11]	2.85	0.88	0.9863
	DLapSRN [11] + Ours	**2.26**	**0.71**	**0.9889**
Super-resolution (RGB guided)	DKN [13]	1.68	0.61	0.9931
	DKN [13] + Ours	**1.59**	**0.59**	**0.9936**

**Table 3:** Quantitative comparison of depth-map reconstruction for different tasks on the Middlebury dataset [37]. Values are given in pixel disparity as provided by the dataset.

Task	Method	RMSE ↓	MAE ↓	SSIM ↑
Denoising	DnCNN [34]	6.21	4.49	0.8932
	DnCNN [34] + Ours	5.55	3.82	**0.9427**
	ADNet [35]	5.32	3.70	0.9200
	ADNet [35] + Ours	**5.09**	**3.44**	0.9383
Deblurring	ResNet [38]	3.06	1.74	0.9574
	ResNet [38] + Ours	**2.96**	**1.67**	**0.9581**
Super-resolution (Depth only)	Bilinear	2.54	1.00	0.9629
	Bilinear + Ours	2.35	0.93	0.9663
	DLapSRN [11]	2.03	0.85	0.9696
	DLapSRN [11] + Ours	**1.69**	**0.74**	**0.9749**
Super-resolution (RGB guided)	DKN [13]	1.23	0.61	0.9805
	DKN [13] + Ours	**1.09**	**0.60**	**0.9806**

## Data Availability

Not applicable.

## Abbreviations

The following abbreviations are used in this manuscript:

CNN	Convolutional neural network
GAN	Generative Adversarial Neural Network
DnCNN	Denoising CNN
ADNet	Attention-guided denoising network
(D)LapSRN	(Depth) Laplacian pyramid super-resolution network
DKN	Deformable kernel network
